# Discordance for Defects in Monochorionic Twins: Prevalence and Impact on Perinatal Outcomes

**DOI:** 10.3390/diagnostics16030385

**Published:** 2026-01-25

**Authors:** Ewelina Litwinska, Izabela Walasik, Monika Szpotanska-Sikorska, Paweł Stanirowski, Tomasz Góra, Tomasz Szajner, Anna Janowicz-Grelewska, Aleksandra Księżopolska, Artur Ludwin, Magdalena Litwinska

**Affiliations:** 1 1st Department of Obstetrics and Gynecology, Medical University of Warsaw, Pl. Starynkiewicza 1/3, 02-015 Warsaw, Poland; ewelina.litwinska@gmail.com (E.L.); izabela.a.walasik@gmail.com (I.W.); monika.szpotanska1@gmail.com (M.S.-S.); stanirowski@gmail.com (P.S.); a.xiesiezopolska@gmail.com (A.K.); ludwin@cm-uj.krakow.pl (A.L.); 2 Clinical Department of Gynecology and Obstetrics, Municipal Hospital, John Paul II, 35-241 Rzeszow, Poland; minddin@gmail.com; 3 Department of Obstetrics and Gynecology, Provincial Clinical Hospital No. 2 Rzeszow, Lwowska 60, 35-301 Rzeszow, Poland; tomissek@gmail.com; 4 Swietokrzyskie Center for the Mother of the Newborn, Specialist Hospital, 25-371 Kielce, Poland; anjangre@gmail.com

**Keywords:** twin pregnancy, monochorionic twins, structural defects

## Abstract

**Background**: Monozygotic twin pregnancies are at increased risk of congenital abnormalities compared to singletons. In 20% of cases, both fetuses are affected (concordance), while in 80% of cases, only one fetus is affected (discordance). This study examines the prevalence of discordance for structural defects in monochorionic (MC) twins, with normal aCGH comparative genomic hybridization (aCGH), reporting the types of detected abnormalities and their possible impact on perinatal outcomes, including the rate of single and double fetal loss before 24 weeks’ gestation and the rate of preterm birth (PB) before 32 weeks’ gestation. **Methods**: This was a retrospective study of discordant structural fetal anomalies in MC twin pregnancies detected at first-trimester scanning in three fetal medicine centers in Poland. **Results**: In the study population of 381 monochorionic twin pregnancies examined at 11–13 weeks’ gestation, 21 (5.5%) pregnancies showed discordant structural defects with normal aCGH result. The most common were cardiac defects (*n* = 8), followed by central nervous system (CNS) (*n* = 6) defects and facial anomalies (*n* = 3). Single or double fetal loss before 28 weeks occurred in four (19%) and two (9%) cases, respectively, and was associated with intertwin crown–rump length (CRL) discordance greater than 20% (*p* = 0.046). PB before 32 weeks’ gestation occurred in nine cases (47%) and was strongly associated with polyhydramnios (*p* = 0.001), which occurred mainly in CNS and facial defects. **Conclusions**: The prevalence of discordant structural defects with normal aCGH results among monochorionic twins is approximately 5%. In pregnancies with discordant defects, cardiac defects are the most common. Intertwin discordance greater than than 20% is associated with an increased risk of fetal demise.

## 1. Introduction

Effective screening for chromosomal abnormalities provided by the measurement of fetal nuchal translucency (NT) thickness has led to the widespread introduction of ultrasound scanning at 11 + 0 to 13 + 6 weeks of gestation [1,2]. During the first-trimester scan, many major fetal abnormalities can be reliably diagnosed [3,4].

**Figure 1:**
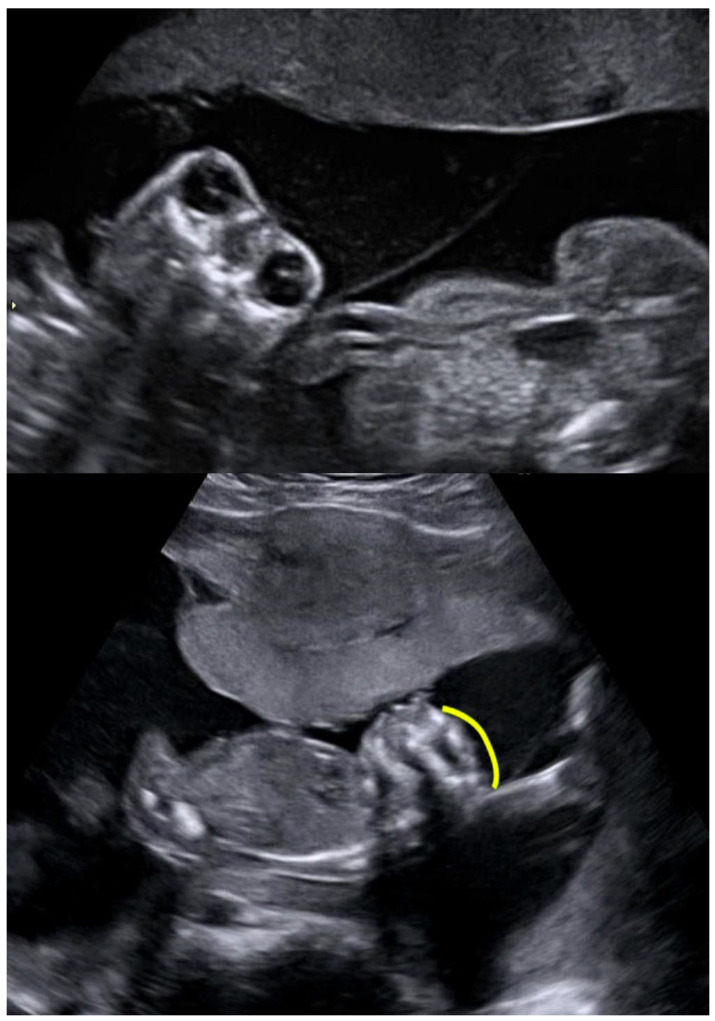
Discordance for anencephaly in MC twins.

In monozygotic twin pregnancies, the prevalence of congenital abnormalities is two times higher than in singletons [5,6]; therefore, the risk of a defect in at least one fetus is four times as high (8%) as in a singleton pregnancy. The prevalence of defects is higher in monoamniotic than in monochorionic diamniotic twins [7]. In 20% of cases, both fetuses are affected (concordance), and in 80% of cases, only one fetus is affected (discordance) [8,9] [Figure 1]. Numerous registry-based studies have confirmed that, compared to singletons, a wide range of birth defects are significantly more common among twins [10,11,12,13,14,15,16]. The most common anomalies in both twins and singletons are cardiovascular anomalies, and the relative risk is higher for twins than singletons. Anomalies of the CNS, the digestive system—in particular, gut atresias—as well as anomalies of GUT and musculoskeletal systems are more common in twins compared to singletons. The rates of chromosomal abnormalities are similar in twins and singletons [5,17].

Our study aimed to examine the prevalence of discordance for structural defects in monochorionic twins with normal aCGH results, to report the types of detected abnormalities, and to assess their potential impact on perinatal outcomes, including the rates of single and double fetal loss before 24 weeks’ gestation and the rate of PB before 32 weeks’ gestation.

## 2. Methods

**Figure 2:**
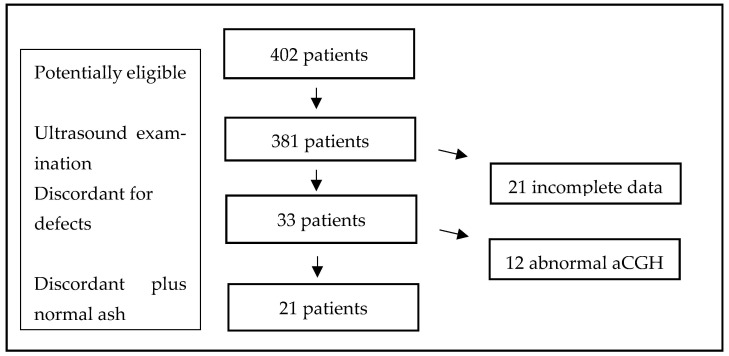
Study population.

This was a retrospective study of 21 monochorionic twin pregnancies diagnosed with discordant structural defects in three fetal medicine centers in Poland between 2019 and 2022. During this time period, 402 cases were identified from perinatal registries created in the participating hospitals. After excluding cases with incomplete data or missing information, 381 pregnancies were selected for analysis. All 381 monochorionic twin pregnancies underwent routine first-trimester combined screening testing for aneuploidies. The combined test was performed by certified sonographers according to the Fetal Medicine Foundation (FMF) algorithm and incorporated the patient’s individual background risk, measurement of fetal nuchal translucency thickness (NT), and maternal serum biomarkers: pregnancy-related plasma protein A (PAPP-A) and free beta-hCG. The anatomy of fetuses was systematically assessed according to the International Society for Ultrasound in Obstetrics and Gynecology (ISUOG) guidelines. If an abnormality was detected, amniocentesis was offered and a sample was taken for analysis separately from each twins. Chorionic villous sampling was not performed in any of these cases. Twelve cases were found to have abnormal aCGH result, and these cases were excluded from the study [Figure 2]. Pregnancies that developed severe TTTS and required fetoscopic laser coagulation of placental anastomoses were also excluded from further analysis.

Databases of participating fetal medicine centers were searched, and cases of monochorionic twins with one structurally normal twin and one twin diagnosed with a major abnormality detected in the first or early second trimester were analyzed. The following data were recorded: amnionicity, type of detected abnormality, discordance in crown-rump length (CRL) between the twins, and the occurrence of adverse perinatal outcomes, including single or double fetal demise before 24 weeks’ gestation and spontaneous PB before 32 weeks’ gestation. CRL discordance was calculated using the following formula: (CRL of the larger twin − CRL of the smaller twin)/CRL of the larger twin × 100%. Selective fetal growth restriction (sFGR) in monochorionic twins was defined as EFW < 10th percentile with intertwin discordance ≥ 25% and classified into type I (persistent positive UA end-diastolic flow), type II (persistent absent/reversed UA end-diastolic flow), and type III (intermittent absent/reversed UA end-diastolic flow). Amniodrainage was performed in cases of severe polyhydramnios with maternal symptoms or cervical shortening, while iatrogenic delivery before 32 weeks was indicated for non-reassuring fetal status, rapid clinical deterioration, or imminent risk of intrauterine demise.

Statistical analysis was performed using STATISTICA 13.3 software (StatSoft Inc., Tulsa, OK, USA). Analyses were conducted to evaluate whether the presence of a specific malformation, crown–rump length (CRL) discordance between twins, selective fetal growth restriction (sFGR), or polyhydramnios was associated with an increased risk of single or double fetal demise (FD) before 24 weeks’ gestation or preterm birth (PTB) before 32 weeks’ gestation.

Categorical variables were compared using the χ^2^ test or Fisher’s exact test, as appropriate, while continuous variables were analyzed using the Mann–Whitney U test. A *p*-value < 0.05 was considered statistically significant.

Due to the small sample size, confidence intervals for key estimates were not calculated, and the results should be interpreted as exploratory. No adjustment for multiple comparisons was applied.

## 3. Results

During the study period, 402 monochorionic pregnancies underwent routine first-trimester screening for chromosomal abnormalities at 11–13 weeks’ gestation. After excluding cases with incomplete data or missing information, 381 pregnancies were included in the analysis.

Structural discordance between twins was identified in 33 pregnancies (8.6%). Of these, 12 were excluded from further analysis because abnormal aCGH results were found in one (*n* = 3) or both twins (*n* = 9). The final study group therefore consisted of 21 pregnancies (5.5%) discordant for structural defects with normal aCGH results in both twins (Figure 2). Among these 21 pregnancies, 17 were monochorionic diamniotic and 4 were monochorionic monoamniotic. The most frequently detected anomalies were congenital heart defects (CHD; *n* = 8), followed by central nervous system (CNS) defects (*n* = 6), facial defects (*n* = 3), abdominal wall defects (*n* = 3), and genitourinary tract (GUT) defects (*n* = 1). Detailed characteristics of these pregnancies are presented in Table 1.

The median gestational age at birth was 33 weeks. Polyhydramnios developed in seven pregnancies (7/21, 33%). Based on cervical shortening below 15 mm, amniodrainage was performed once in cases 15 and 17 and twice in cases 1, 3, and 6. Cervical cerclage was not performed in any case.

Selective fetal growth restriction (sFGR) was diagnosed in six pregnancies (6/21, 29%), one of which required iatrogenic preterm birth before 32 weeks’ gestation (Table 2).

Single fetal demise occurred in four pregnancies (4/21, 19%), while double fetal demise occurred in two cases (2/21, 9%). The rates of fetal demise did not differ significantly between defect categories. In five of the six pregnancies complicated by single or double fetal demise, the discordance in crown–rump length (CRL) at the 11–13-week scan exceeded 20% (5/6, 83%), which was statistically significant (*p* = 0.046).

After excluding cases with double fetal demise (*n* = 2), preterm birth before 32 weeks occurred in 9 of 19 pregnancies (47%). In seven of these nine cases (78%), preterm birth occurred in pregnancies complicated by polyhydramnios and resulted from premature rupture of membranes (*p* = 0.001). CNS and facial defects were the most frequent anomalies in this subgroup (5/7, 71%).

An exploratory analysis of factors associated with adverse perinatal outcomes is presented in Table 3.

## 4. Discussion

### 4.1. Main Findings of the Study

Our study demonstrates a higher prevalence of specific malformations in MC twins compared with DC twins and singletons. The most common defects were CHD, followed by CNS, facial, abdominal wall, and GUT anomalies. Among CHD, VSD and AVSD were most frequent. The diagnosis of a CHD did not increase the risk of miscarriage or PB. Instead, single or double FD was more closely associated with large intertwin discordance than with defect type. In MC twin pregnancies, the presence of a defect causing polyhydramnios was strongly associated with PB before 32 weeks’ gestation.

### 4.2. Comparison with Previous Studies

In our cohort, CHD accounted for 38% of all anomalies. Previous studies consistently report an increased risk of CHD in twins, with MC twins at higher risk than DC twins. For example, Best et al. analyzed 399,414 singleton births and 11,871 twin births and found a 73% increased risk of CHD in twins compared with singletons. The risk was 172% in MC twins and 49% in DC twins compared with singletons [18].

In our study, pregnancies complicated by TTTS were excluded, as TTTS predisposes to certain CHD, mainly pulmonary stenosis. Nevertheless, non-TTTS MC twins remain at increased risk of CHD, and fetal echocardiography should be considered for all MC twin pregnancies [19].

Miscarriage or FD between 12 and 23 weeks’ gestation occurs in ~1% of singletons, 2% of DC twins, and 12% of MC twins [20]. In our cohort, single or double FD before 24 weeks’ gestation occurred in six cases (25%) and did not differ significantly between defect categories. Notably, 83% of these cases had a CRL discordance > 20%, consistent with previous reports showing an increased risk of FD in MC twins with CRL discordance > 15% [21].

The risk of spontaneous delivery between 24 and 32 weeks’ gestation is ~1% in singletons, 6% in DC twins, and 9% in MC twins [22]. In our study, spontaneous PB occurred in 47% of cases, most frequently in pregnancies complicated by polyhydramnios. CNS and facial anomalies may potentially impair fetal swallowing, which could influence amniotic fluid dynamics; however, this hypothesis could not be directly evaluated due to the small sample size.

All cases considered for amniodrainage had a single deepest pocket >8 cm; however, only pregnancies with a short cervix and/or maternal symptoms underwent intervention. These data, along with cervical examination, may inform the indication for therapeutic amniodrainage. Given that nearly half of the patients delivered preterm, antenatal corticosteroid therapy should be considered on an individual basis.

### 4.3. Clinical Implication and Selective Fetocide

In our cohort, pregnancies with discordant defects were managed expectantly. Selective fetocide may be considered in cases where the abnormal twin threatens the survival of the co-twin. In MC pregnancies, placental vascular anastomoses connecting the two fetal circulations pose significant risks: drugs injected into the affected twin may reach the healthy twin, causing immediate demise. Additionally, acute hemorrhage from the survivor into the dying fetus may result in organ damage. Therefore, any selective reduction must ensure complete and permanent occlusion of both arterial and venous blood flow in the umbilical cord of the affected twin.

Several techniques have been described, with the most commonly used methods being bipolar cord coagulation (BCO) [23,24,25,26,27], radiofrequency ablation (RFA) [28,29,30,31,32,33], and intrafetal laser (IL) [34]. These approaches require careful consideration of risks and benefits.

### 4.4. Strengths and Limitations of the Study

The strength of the study is its substantial contribution to the limited number of MC twin pregnancies discordant for structural defects managed expectantly. The added value is invasive testing performed in both twins, which included not only karyotype but also aCGH. This approach enables the detection of specific variants, such as gene deletions or duplications.

The major limitations of the study are related to its retrospective design and the small sample size. These limitations result from the rarity of the condition.

## 5. Conclusions

Monochorionic twins are at high risk for structural abnormalities. In cases of discordant defects, the most common are cardiac defects. Intertwin discordance of more than 20%, rather than type of a defect, increases the risk of single or double fetal demise.

Polyhydramnios, which occurred mainly in CNS and facial defects, significantly increases the risk of PB before 32 weeks’ gestation.

### Implications for Clinical Practice

The prevalence of defects is substantially higher in monochorionic twins compared with dichorionic twins and singletons. Therefore, special attention should be paid when examining monochorionic twins. CHD are more common in both non-TTTS and TTTS twins; therefore, all monochorionic twins should be offered fetal echocardiography.

In cases of defects associated with polyhydramnios, which significantly increases the risk of PB before 32 weeks’ GA, early intervention, including selective fetocide, should be considered as a means to reduce the risk of prematurity.

## Figures and Tables

**Table 1:** Individual data of pregnancies included in the analysis.

Case Number	System Affected	Type of a Defect	Time of Diagnosis	Discordance in CRL	Amnionicity
1	CNS	Anencephaly	11–13 weeks	13%	Diamniotic
2	CNS	Spina bifida	11–13 weeks	5%	Diamniotic
3	CNS	Anencephaly	11–13 weeks	19%	Diamniotic
4	CNS	Encephalocele	11–13 weeks	12%	Diamniotic
5	CNS	Spina bifida	16 weeks	10%	Diamniotic
6	CNS	Holoprosencephaly	11–13 weeks	13%	Diamniotic
7	CHD	VSD, CoA	11–13 weeks; CoA 27 weeks	26%	Diamniotic
8	CHD	VSD	11–13 weeks	24%	Diamniotic
9	CHD	AVSD	11–13 weeks	30%	Monoamniotic
10	CHD	VSD	11–13 weeks	24%	Diamniotic
11	CHD	ToF	11–13 weeks	23%	Diamniotic
12	CHD	ToF	11–13 weeks	11%	Monoamniotic
13	CHD	AVSD	11–13 weeks	17%	Diamniotic
14	CHD	VSD	11–13 weeks	21%	Diamniotic
15	Facial	Cleft	11–13 weeks	8%	Diamniotic
16	Facial	Cleft	11–13 weeks	18%	Diamniotic
17	Facial	Cleft	16 weeks	22%	Diamniotic
18	Abdominal	Omphalocele	11–13 weeks	30%	Monoamniotic
19	Abdominal	Omphalocele	11–13 weeks	10%	Diamniotic
20	Abdominal	Omphalocele	11–13 weeks	28%	Diamniotic
21	GUT	Unilateral renal agenesis	16 weeks	4%	Monoamniotic

**Table 2:** Outcomes of pregnancies included in the study.

Case Number	Type of a Defect	Discordance in CRL > 20%	Complications	Interventions	Single/Double FD	GA at Birth
1	Anencephaly	No	Polyhydramions, PPROM	Amniodrainage (2×)	No	31 (spontaneous, PPROM)
2	Spina bifida	No	-		No	35
3	Anencephaly	No	Polyhydramions, PPROM	Amniodrainage (2×)	Yes (single)	29 (spontaneous, PPROM)
4	Encephalocele	No	-		No	36
5	Spina bifida	No	-		No	36
6	Holoprosencephaly	No	Polyhydramions, PPROM	Amniodrainage (2×)	No	31 (spontaneous, PPROM)
7	VSD, CoA	Yes	sFGR		No	31 (iatrogenic: sFGR)
8	VSD	Yes	sFGR		No	33
9	AVSD	Yes	-		Yes (double)	16
10	VSD	Yes	sFGR		Yes (single)	34
11	ToF	Yes	sFGR		No	34
12	ToF	No	-		No	32 (iatrogenic, MCMA twins)
13	AVSD	No	-		No	36
14	VSD	Yes	-		No	33
15	Cleft	No	Polyhydramnios	Amniodrainage (1×)	No	28 (spontaneous, PPROM)
16	Cleft	No	Polyhydramnios		No	30 (spontaneous, PPROM)
17	Cleft	Yes	Polyhydramnios, sFGR	Amniodrainage (1×)	Yes (single)	31 (spontaneous, PPROM)
18	Omphalocele	Yes	Double FD		Yes (double)	16
19	Omphalocele	No	-		No	35
20	Omphalocele	Yes	sFGR Polyhydramions		Yes (single)	30 (spontaneous, PPROM)
21	Unilateral renal agenesis		-		No	31

**Table 3:** Analysis of possible factors associated with adverse perinatal outcomes.

Factor Affecting Perinatal Outcomes	Single/Double FD < 24 Weeks	*p*-Value	PTB < 32 Weeks	*p*-Value < 0.05 Was Considered Statistically Significant
Group of defects				
CNS (*n* = 6)	1/6	0.623	3/6	1.00
CHD (*n* = 8)	2/8	1.00	2/8 (iatrogenic)	0.379
Facial (*n* = 3)	1/3	1.00	3/3	0.09
Abdominal (*n* = 3)	2/3	1.184	1/3	1.00
GUT (*n* = 1)	0/1	1.0	0/1	1.00
Discordance of CRL > 20% (*n* = 9)	5/9	0.046 *	3/9	0.387
sFGR (*n* = 6)	3/6	0.291	2/6	0.635
Polyhydramnios (*n* = 7)	3/7	0.354	7/7	0.001 *

Statistically significant results are marked *.

## Data Availability

The data presented in this study are available on request from the corresponding author due to institutional policies, data protection regulations, and the need to ensure that data sharing is for approved academic, non-commercial research purposes.

## Abbreviations

NT	Nuchal translucency
FMF	Fetal Medicine Foundation
PAPP-A	Pregnancy related plasma protein A
ISUOG	International Society for Ultrasound in Obstetrics and Gynecology
CRL	Crown-rump length
TTTS	Twin to twin transfusion syndrome
CNS	Central Nervous System
CHD	Congenital Heart Defect
GUT	Genitourinary defect
VSD	Ventriculoseptal defect
CoA	Coarctation of aorta
ToF	Tetralogy of Fallot
FD	Fetal demise
GA	Gestational age at birth
S	Spontaneous preterm birth
I	Iatrogenic preterm birth
IL	Interstitial laser
PB	Preterm birth
RFA	Radiofrequency ablation
BCO	Bipolar cord occlusion
aCGH	Array comparative genomic hybridization

## References

[1] Nicolaides K.H., Azar G.B., Byrne D., Mansur C.A., Marks K. (1992). Nuchal translucency: Ultrasound screening for chromosomal defects in the first trimester of pregnancy. Br. Med. J..

[2] Snijders R.M.J., Noble P., Sebire N., Souka A., Nicolaides K.H. (1998). UK multicentre project on assessment of risk of trisomy 21 by maternal age and fetal nuchal translucency thickness at 10–14 weeks of gestation. Lancet.

[3] Souka A.P., Nicolaides K.H. (1997). Diagnosis of fetal abnormalities at the 10–14-week scan. Ultrasound Obstet. Gynecol..

[4] Syngelaki A., Cimpoca B., Litwinska E., Akolekar R., Nicolaides K.H. (2020). Diagnosis of fetal defects in twin pregnancies at routine 11–13-week ultrasound examination. Ultrasound Obstet. Gynecol..

[5] Glinianaia S.V., Rank J., Wright C. (2008). Congenital anomalies in twins: A register-based study. Hum. Reprod..

[6] Yu Y., Cozen W., Hwang A.E., Cockburn M.G., Zadnick J., Hamilton A.S., Mack T., Figueiredo J.C. (2018). Birth Anomalies in Monozygotic and Dizygotic Twins: Results From the California Twin Registry. J. Epidemiol..

[7] Baxi L.V., Walsh C.A. (2010). Monoamniotic twins in contemporary practice: A single-center study of perinatal outcomes. J. Matern. Fetal Neonatal Med..

[8] Gul A., Cebeci A., Aslan H., Polat I., Sozen I., Ceylan Y. (2005). Perinatal outcomes of twin pregnancies discordant for major fetal anomalies. Fetal Diagn. Ther..

[9] Weber M.A., Sebire N.J. (2010). Genetics and developmental pathology of twinning. Semin. Fetal Neonatal Med..

[10] Dawson A.L., Tinker S.C. (2016). the National Birth Defects Prevention Study. Twinning and major birth defects, National Birth Defects Prevention Study, 1997–2007. J. Epidemiol. Community Health.

[11] Rider R.A., Stevenson D.A., Rinsky J.E., Feldkamp M.L. (2013). Association of twinning and maternal age with major structural birth defects in Utah, 1999 to 2008. Birth Defects Res. A Clin. Mol. Teratol..

[12] Ramos-Arroyo M.A. (1991). Birth defects in twins: Study in a Spanish population. Acta Genet. Med. Gemellol..

[13] Zhang X.H., Qiu L.Q., Huang J.P. (2011). Risk of birth defects increased in multiple births. Birth Defects Res. A Clin. Mol. Teratol..

[14] Li S.J., Ford N., Meister K., Bodurtha J. (2003). Increased risk of birth defects among children from multiple births. Birth Defects Res. A Clin. Mol. Teratol..

[15] Mastroiacovo P., Castilla E.E., Arpino C., Botting B., Cocchi G., Goujard J., Marinacci C., Merlob P., Métneki J., Mutchinick O. (1999). Congenital malformations in twins: An international study. Am. J. Med. Genet..

[16] Tang Y., Ma C.X., Cui W., Chang V., Ariet M., Morse S.B., Resnick M.B., Roth J. (2006). The risk of birth defects in multiple births: A population-based study. Matern. Child Health J..

[17] Kroeldrup L., Larsen L.A., Fagerberg C., Hertz J.M., Christensen K. (2017). Chromosomal Aberrations in Monozygotic and Dizygotic Twins Versus Singletons in Denmark During 1968–2009. Twin Res. Hum. Genet..

[18] Best K.E., Rankin J. (2015). Increased risk of congenital heart disease in twins in the North of England between 1998 and 2010. Heart.

[19] Bahtiyar M.O., Dulay A.T., Weeks B.P., Friedman A.H., Copel J.A. (2007). Prevalence of congenital heart defects in monochorionic/diamniotic twin gestations: A systematic review. J. Ultrasound Med..

[20] Rustico M.A., Baietti M.G., Coviello D., Orlandi E., Nicolini U. (2005). Managing twins discordant for fetal anomaly. Prenat. Diagn..

[21] Litwinska E., Syngelaki A., Cimpoca B., Sapantzoglou I., Nicolaides K.H. (2020). Intertwin discordance in fetal size at 11–13 weeks’ gestation and pregnancy outcome. Ultrasound Obstet. Gynecol..

[22] Sebire N.J., Snijders R.J.M., Hughes K., Sepulveda W., Nicolaides K.H. (1997). The hidden mortality of monochorionic twin pregnancies. Br. J. Obstet. Gynaecol..

[23] Deprest J.A., Audibert F., Van Schoubroeck D., Hecher K., Mahieu-Caputo D. (2000). Bipolar coagulation of the umbilical cord in complicated monochorionic twin pregnancy. Am. J. Obstet. Gynecol..

[24] Taylor M.J.O., Shalev E., Tanawattancharoen S., Jolly M., Kumar S., Weiner E. (2002). Ultrasound guided umbilical cord occlusion using bipolar diathermy for stage III/IV twin-twin transfusion syndrome. Prenat. Diagn..

[25] Robyr R., Yamamoto M., Ville Y. (2005). Selective fetocide in complicated monochorionic twin pregnancies using ultrasound-guided bipolar cord coagulation. BJOG.

[26] Lewi L., Gratacos E., Ortibus E., Van Schoubroeck D., Carreras E., Higueras T., Perapoch J., Deprest J. (2006). Pregnancy and infant outcome of 80 consecutive cord coagulations in complicated monochorionic multiple pregnancies. Am. J. Obstet. Gynecol..

[27] Roman A., Papanna R., Johnson A., Hassan S.S., Moldenhauer J., Molina S., Moise K.J. (2010). Selective reduction in complicated monochorionic pregnancies: Radiofrequency vs. bipolar cord coagulation. Ultrasound Obstet. Gynecol..

[28] Moise K.J., Johnson A., Moise K.Y., Nickeleit V. (2008). Radiofrequency ablation for selective reduction in the complicated monochorionic gestation. Am. J. Obstet. Gynecol..

[29] Cabassa P., Fichera A., Prefumo F., Taddei F., Gandolfi S., Maroldi R., Frusca T. (2013). The use of radiofrequency in the treatment of twin reversed arterial perfusion sequence: A case series and review of the literature. Eur. J. Obstet. Gynecol. Reprod. Biol..

[30] Lee H., Wagner A.J., Sy E., Ball R., Feldstein V.A., Goldstein R.B., Farmer D.L. (2007). Efficacy of radiofrequency ablation for twin-reversed arterial perfusion sequence. Am. J. Obstet. Gynecol..

[31] Lu J., Ting Y.H., Law K.M., Lau T.K., Leung T.Y. (2013). Radiofrequency ablation for selective reduction in complicated monochorionic multiple pregnancies. Fetal Diagn. Ther..

[32] Berg C., Holst D., Mallmann M.R., Gottschalk I., Gembruch U., Geipel A. (2014). Early vs late intervention in twin reversed arterial perfusion sequence. Ultrasound Obstet. Gynecol..

[33] Kumar S., Paramasivam G., Zhang E., Jones B., Noori M., Prior T., Vasudeva A., Wimalasundera R.C. (2014). Perinatal- and procedure-related outcomes following radiofrequency ablation in monochorionic pregnancy. Am. J. Obstet. Gynecol..

[34] Chaveeva P., Peeva G., Pugliese S.G., Shterev A., Nicolaides K.H. (2017). Intrafetal laser ablation for embryo reduction from dichorionic triplets to dichorionic twins. Ultrasound Obstet. Gynecol..

